# Causal relationship between plasma lipidome and rosacea: a Mendelian randomization analysis

**DOI:** 10.3389/fendo.2025.1427656

**Published:** 2025-04-28

**Authors:** Xiaoxue Wang, Zexin Zhu

**Affiliations:** ^1^ Department of Dermatology, The Second Affiliated Hospital of Xi’an Jiaotong University, Xi’an, China; ^2^ Department of Surgical Oncology, The Comprehensive Breast Care Center, The Second Affiliated Hospital of Xi’an Jiaotong University, Xi’an, China

**Keywords:** plasma lipidome, rosacea, Mendelian randomization, causal inference, protective

## Abstract

**Background:**

Rosacea is a common chronic inflammatory skin disease. Limited studies reported the association between plasma lipidome and rosacea.

**Methods:**

We employed a two-sample Mendelian randomization (MR) study to assess the causality between plasma lipidome and rosacea. Plasma lipidome association genome-wide association study (GWAS) data were collected. The inverse variance weighted (IVW) method was utilized as the principal method in our Mendelian randomization (MR) study; we also used the MR-Egger, weighted median, simple mode, and weighted mode methods. The MR-Egger intercept test, Cochran’s Q test, MR-Pleiotropy RESidual Sum and Outlier (MR-PRESSO), and leave-one-out analysis were conducted to identify heterogeneity and pleiotropy.

**Results:**

A total of 179 lipid species were analyzed; among them, five lipid species were closely related to rosacea. Two species of sterol ester [sterol ester (27:1/22:6) and sterol ester (27:1/15:0)], two species of phosphatidylethanolamine [phosphatidylethanolamine (O-18:2_20:4) and phosphatidylethanolamine (18:0_20:4)], and one species of sphingomyelin [sphingomyelin (d34:0)] were causally associated with rosacea (*P* < 0.05). All of them play protective roles in patients with rosacea. No heterogeneity or pleiotropy was observed.

**Conclusion:**

This study provided new evidence of the relationship between plasma lipidome and rosacea. Our MR suggested that five lipid species play protective roles in rosacea progression. These could be novel and effective ways to treat rosacea.

## 1 Introduction

Rosacea is a prevalent chronic inflammatory dermatological condition that primarily impacts the cheeks, chin, nose, forehead, and ocular regions (1, 2). The reported prevalence of rosacea varies significantly, ranging from 1% to 22%, a variation attributed to geographical and demographic factors (3, 4). A recent systematic review estimated the global prevalence of rosacea to be approximately 5.5% among the adult population (5). Contrary to earlier studies that indicated a higher prevalence in females (1, 2), the findings of this systematic review suggest that both men and women are equally affected by the condition (5). The pathophysiology of rosacea remains inadequately understood. Mechanistically, the pathogenesis of rosacea is associated with various inflammatory pathways, which involve the dysregulation of both the innate and adaptive immune systems (2, 6). Investigations into single nucleotide polymorphisms (SNPs) in genes linked to rosacea indicate that genetic factors may also play a role (7). Factors such as stress, ultraviolet radiation, consumption of spicy foods, smoking, and alcohol intake have been identified as potential exacerbators of symptoms (1). The diagnosis of rosacea is primarily based on clinical manifestations and skin biopsy findings (1). Treatment options for rosacea include skin care regimens, topical medications such as brimonidine and ivermectin (8), oral antibiotics like doxycycline and minocycline (9), as well as biologic agents such as Secukinumab (10) and Erenumab (2). It is important to note that rosacea is a chronic condition; while patients may experience periods of remission due to various treatments, relapses are frequently observed (1).

Plasma lipids, including high-density lipoprotein cholesterol (HDL-C), low-density lipoprotein cholesterol (LDL-C), triglycerides (TG), and total cholesterol (TC), are routinely assessed and have been established as significant risk factors for various health conditions, particularly cardiovascular disease (CVD). Recent studies have expanded our comprehension of circulating lipid diversity by identifying additional lipid species, such as cholesterol esters (CE), lysophosphatidylcholines (LPC), phosphatidylcholines (PC), phosphatidylethanolamines (PE), and sphingomyelins (SM) (11).

Several studies reported the relationship between plasma lipids and skin disease. For instance, a significant reduction in serum high-density lipoprotein cholesterol (HDL-C) levels has been documented in patients with chronic spontaneous urticaria (12); on the other hand, patients suffering from atopic dermatitis exhibited a notable decrease in cholesteryl esters, free cholesterol, lysophosphatidylcholine (particularly the 16:0 species), and phosphatidylethanolamine (13). Additionally, adolescents diagnosed with atopic dermatitis (AD) within the Asian demographic demonstrated significantly elevated levels of total cholesterol (TC) and low-density lipoprotein cholesterol (LDL-C).

Mendelian randomization (MR) utilizes one or more genetic variants as instrumental variables (IVs) based on genome-wide association studies (GWAS). MR studies can infer the causal effects of exposure on an outcome. Recently, MR analysis also reported the causal relationship between lipids and skin diseases. For instance, MR analysis showed that HDL deficiency and high LDL-C and TG have a causal relationship with incident psoriasis genetically (14, 15). To our knowledge, no study has yet investigated the causal effect of plasma lipidome on the risk of rosacea using Mendelian randomization. Our investigation aimed to explore the plasma lipidome risk variants as instrumental variables for rosacea utilizing two-sample MR.

## 2 Materials and methods

### 2.1 Study design

**Figure 1:**
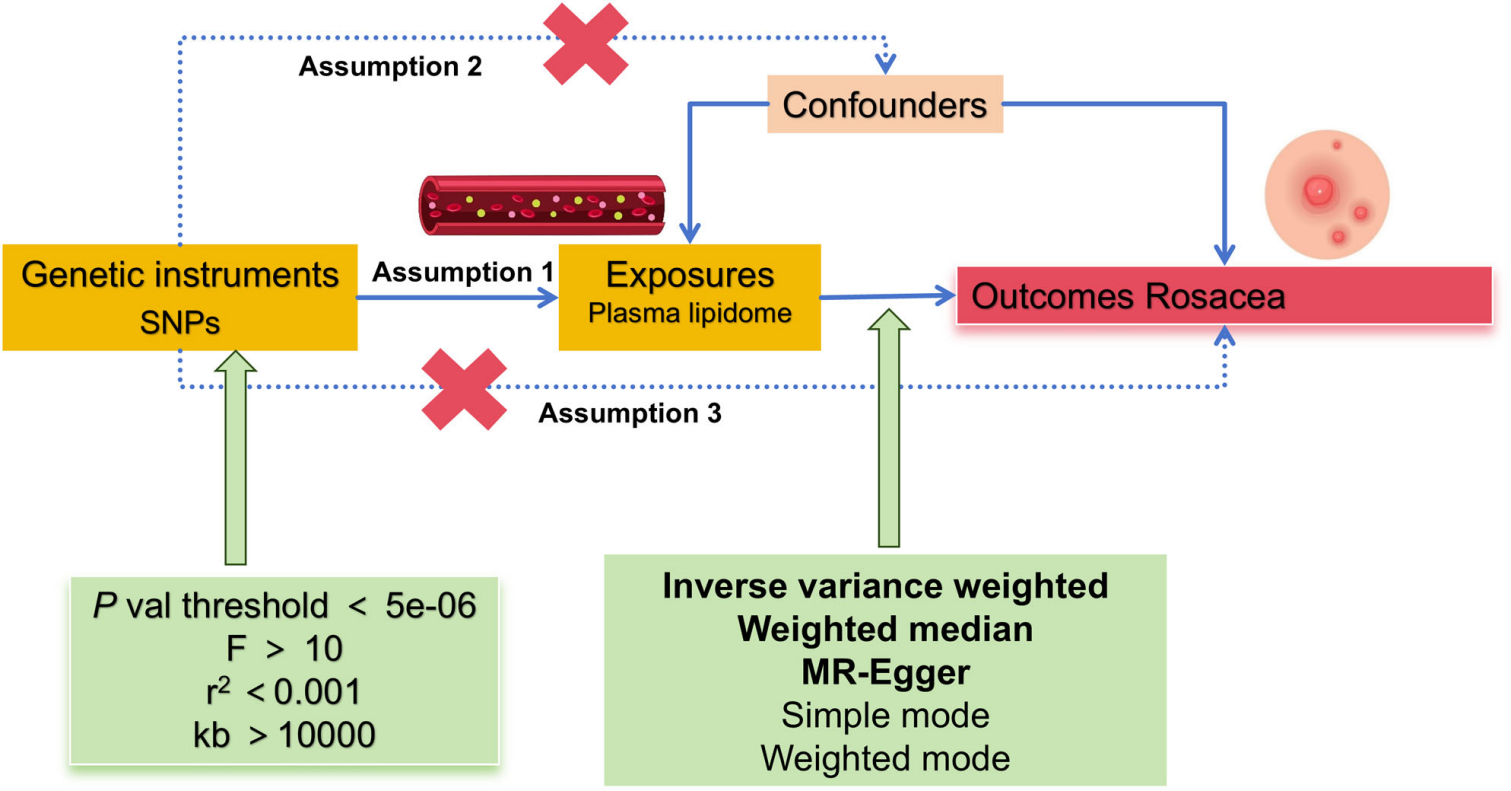
Flowchart schematic diagram followed by the MR analysis’s principle of this study.

According to the MR framework (**Figure 1**), three key assumptions are included (1): Relevance Assumption: Single nucleotide polymorphisms (SNPs) that are substantially linked to exposures are used as instrumental variables (IVs). (2) Independence Assumption: These SNPs (IVs) should not show any correlation with the relevant confounding factor. (3) Exclusivity Assumption: These SNPs (IVs) should affect outcomes only through its effect on exposure (16, 17).

### 2.2 Data sources

The plasma lipidome GWAS data were obtained from the prospective GeneRISK cohort including 7,174 individuals (18), summarized by Ottensmann L et al. (11). A total of 179 lipid species [GWAS Catalog (https://www.ebi.ac.uk/gwas/, GCST90277238–GCST90277416)] belonging to 13 lipid classes covering four major lipid categories (glycerolipids, glycerophospholipids, sphingolipids, and sterols) were detected. The GWAS data related to rosacea was obtained from the IEU OpenGWAS project, GWAS ID: finn-b-L12_ROSACEA, which included 1,195 cases and 211,139 controls, featuring 16,380,452 SNPs, with the study population being of European descent. All participants provided informed written consent, and all studies were reviewed and approved by institutional ethics review committees at the involved institutions.

### 2.3 Instrumental variables selection

Related IVs (plasma lipidome) for MR analysis followed particular principles: SNPs should be associated with exposures at the locus-wide significance level: *P* < 5e−06. In addition, linkage disequilibrium (LD) coefficient r^2^ should be less than 0.001, not closely related (clumping window more than 10,000 kb) to ensure exposure instrument independence. The F statistic was employed to assess the strength of the IVs, with values exceeding 10, thereby suggesting the absence of weak instrumental variable bias. The F-value is calculated using the formula F = R^2^(N − 2)/(1 − R^2^), where R² denotes the proportion of variance accounted for by SNPs in the exposure dataset, and N represents the sample size of the GWAS (16, 17).

### 2.4 MR analysis

Causal associations between plasma lipidome and rosacea were determined using MR analysis. In the exposure–outcome analysis, we employed MR with more than two SNPs serving as IVs. Our MR analysis used each of the five methods: inverse variance weighted (IVW) was performed as the primary statistical analysis method in our MR analysis for evaluating causal effects, with additional methodologies, namely, simple mode, weighted median, weighted mode, and MR-Egger, being utilized to further corroborate the findings. The MR-Egger method is implemented through a straightforward modification of the weighted linear regression technique previously outlined. MR-Egger was specifically employed to evaluate the robustness of the MR results as a form of validation (16, 17, 19).

The heterogeneity of the chosen SNPs was evaluated using Cochrane’s Q test, where a *P*-value of more than 0.05 suggested the lack of heterogeneity. The random effects model was used once significant heterogeneity has been identified. We evaluated the possible bias from horizontal pleiotropy using the weighted median and MR-Egger regression in order to gauge the robustness of the IVW method. The MR-PRESSO (MR-Pleiotropy RESidual Sum and Outlier) test was used to appraise outliers that might have been influenced by horizontal pleiotropy. The causal-effect estimates for individual variants were displayed using a scatter plot. Thereafter, we performed a leave-one-out analysis to examine the stability of the results in the context of a single SNP’s influence and presented the findings in a forest plot (16, 17, 19).

### 2.5 Statistical analysis

All statistical analysis were conducted in R software (Version 4.3.2) using the TwoSampleMR package (Version 0.5.8). The statistical significance level is *P* <0.05. Pooled odds ratio (OR) with 95% confidence interval (CI) were calculated. The IVW method was primarily employed to evaluate the causal relationships between 179 lipid species and rosacea, with the findings illustrated through a volcano plot; significant results were subsequently represented using a forest plot. The false discovery rate (FDR) correction was applied to adjust all *P*-value thresholds, whereby *P*-values exceeding the FDR-corrected threshold but remaining below 0.05 were regarded as indicative of potential causal associations.

## 3 Results

### 3.1 MR analysis

**Figure 2:**
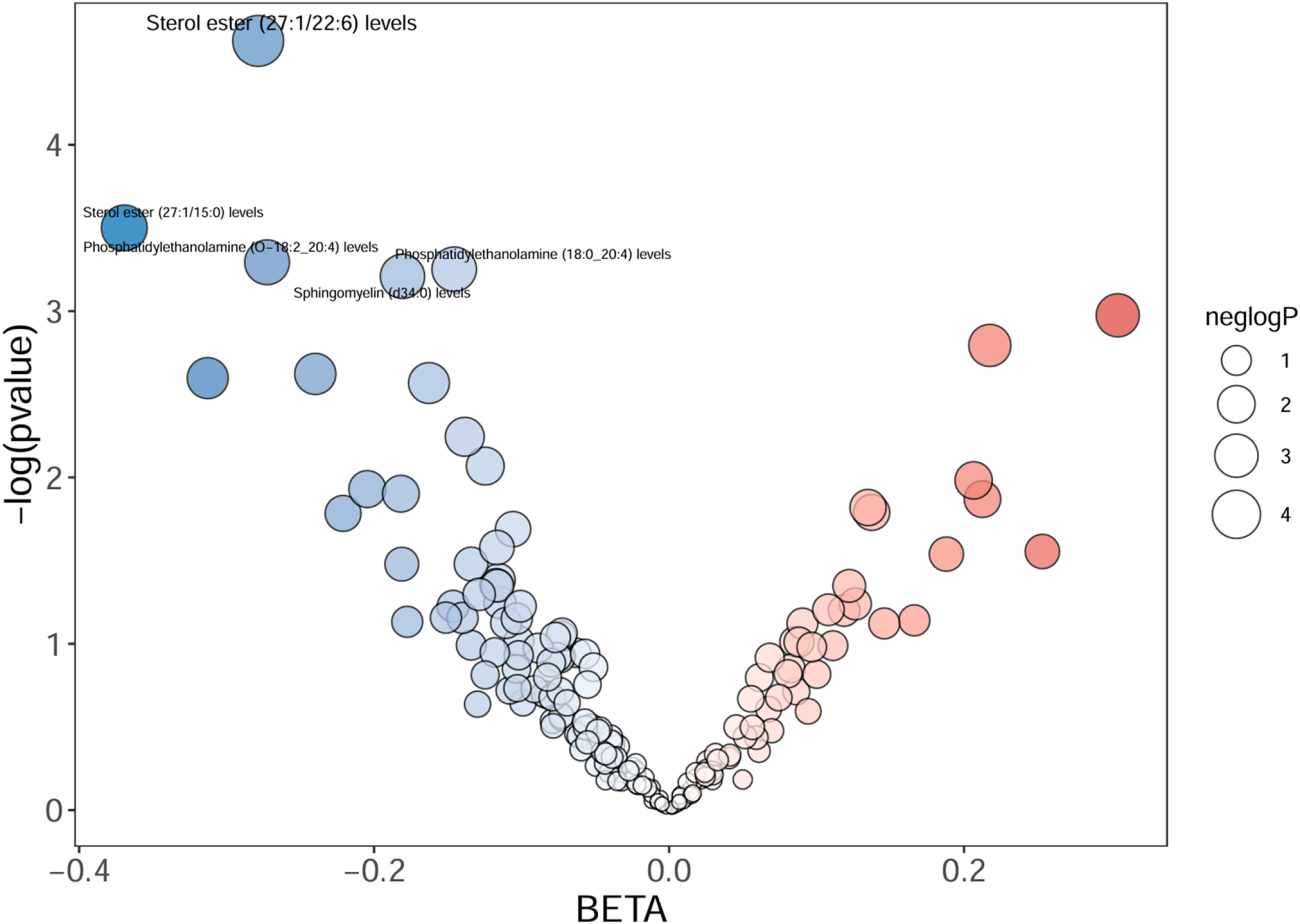
The volcano plot shows the association between 179 lipid species and rosacea risk. The X-axis represents the β value, and the Y-axis shows the logarithmic *p*-value in base 10. Sterol ester (27:1/22:6), sterol ester (27:1/15:0), phosphatidylethanolamine (O-18:2_20:4), phosphatidylethanolamine (18:0_20:4), and sphingomyelin (d34:0) indicate the *P*-value <0.05.

**Figure 3:**
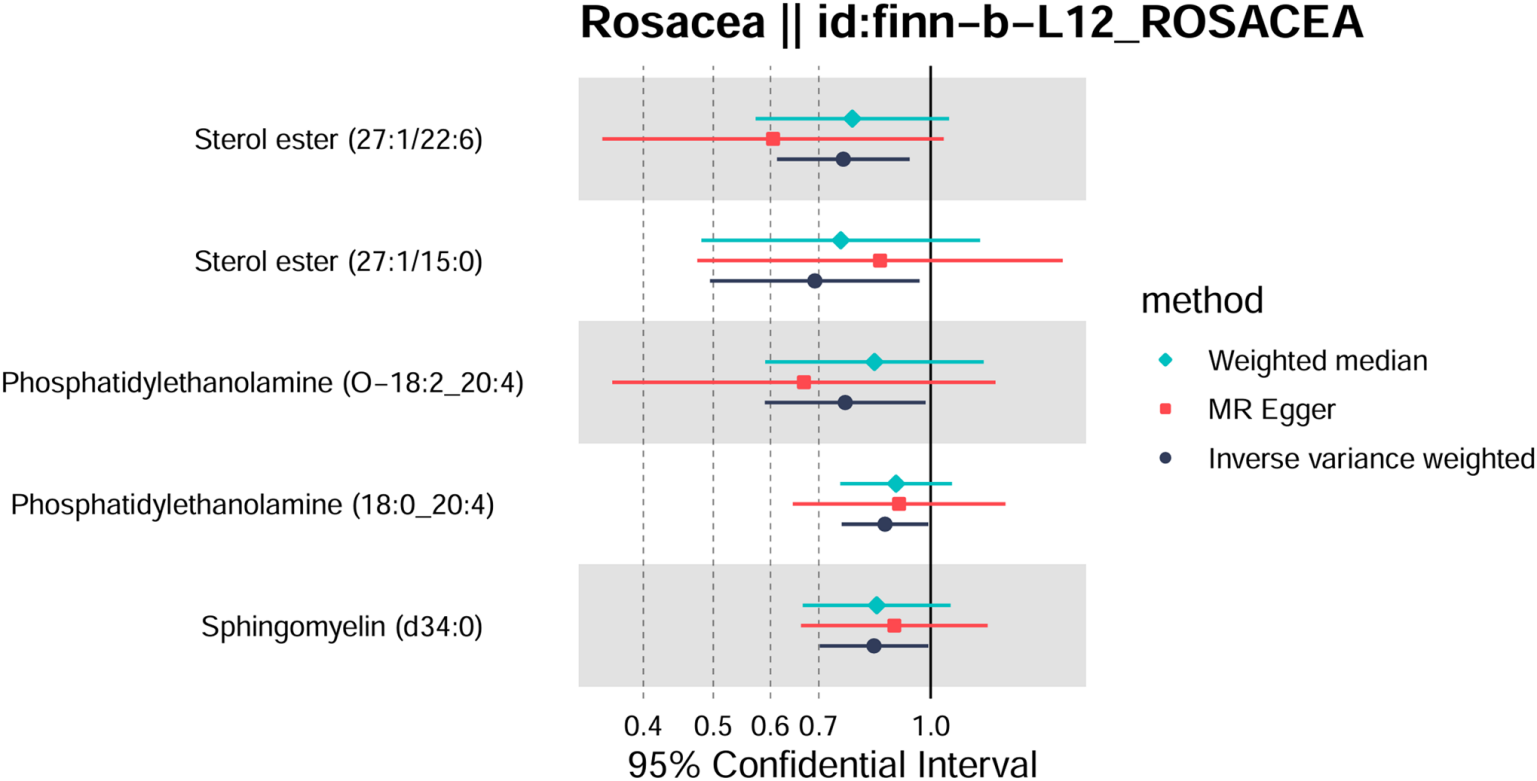
Forest plot of Mendelian randomization analysis for sterol ester (27:1/22:6), sterol ester (27:1/15:0), phosphatidylethanolamine (O-18:2_20:4), phosphatidylethanolamine (18:0_20:4), sphingomyelin (d34:0), and rosacea risk. The results of inverse variance weighted (IVW), weighted median, and MR-Egger are shown.

**Figure 4:**
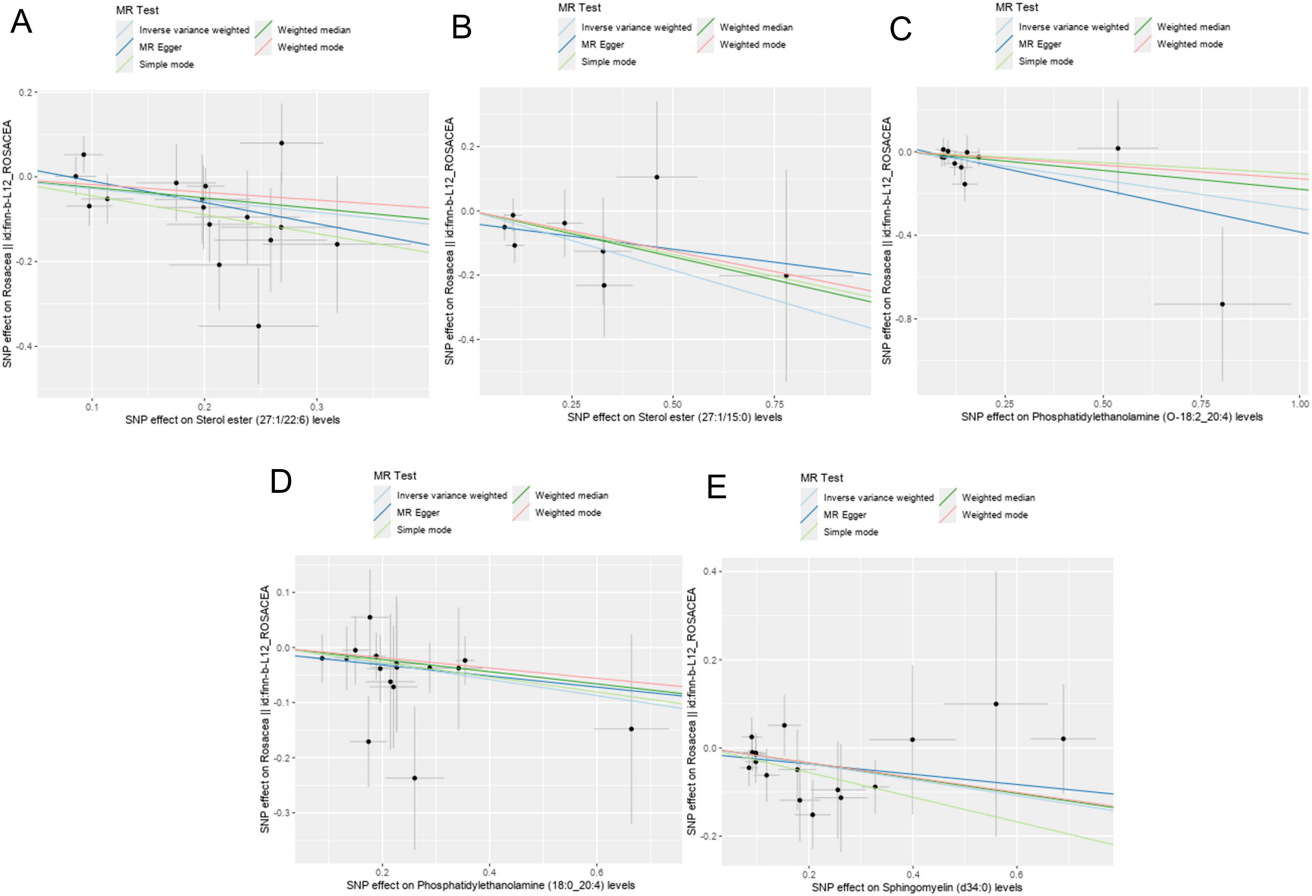
Scatter plots showing significant causal effects between plasma lipidome and rosacea. **(A)** Sterol ester (27:1/22:6). **(B)** Sterol ester (27:1/15:0). **(C)** Phosphatidylethanolamine (O-18:2_20:4). **(D)** Phosphatidylethanolamine (18:0_20:4). **(E)** Sphingomyelin (d34:0).

Totally, we analyzed the plasma lipidome (1,893 SNPs, detailed in **Supplementary Table S1**) for their causal association with rosacea. As mentioned, the inverse variance weighted (IVW) method was chosen as the primary statistical analysis method. MR analysis revealed that among the 179 lipid species, according to the results of the IVW method (*P* < 0.05, **Figure 2**), five lipid species exhibited a significant association with the outcome variable of rosacea. Notably, all of these lipid species demonstrated an odds ratio (OR) of less than 1 (**Figure 3**, detailed in **Table 1**). Among them, two species of sterol ester [sterol ester (27:1/22:6) (OR = 0.757, 95% CI = 0.613–0.935, *P* = 0.01) and sterol ester (27:1/15:0) (OR = 0.691, 95% CI = 0.495–0.965, *P* = 0.03] resulted in a protective factor for rosacea; two species of phosphatidylethanolamine [phosphatidylethanolamine (O-18:2_20:4) (OR = 0.761, 95% CI = 0.589–0.984, *P* = 0.03) and phosphatidylethanolamine (18:0_20:4) (OR = 0.864, 95% CI = 0.753–0.992, *P* = 0.03)] showed a protective effect on rosacea; one species of sphingomyelin [sphingomyelin (d34:0) (OR = 0.835, 95% CI = 0.702–0.992, *P* = 0.04)] also resulted in a causal protective relationship with rosacea. The scatter plots for the causal relationship between plasma lipidome and rosacea are presented in **Figure 4**. It is noteworthy that all five lipid species exhibited a negative correlation with rosacea, indicating that these lipid types may have a causal protective effect against the condition. A detailed analysis of the components of each lipid species is provided in the **Supplementary Materials**.

**Table 1:** Causal relationship between the plasma lipidome and rosacea.

Exposure	Methods	OR	Low 95% CI	Up 95% CI	*P*
	Inverse variance weighted	0.757	0.613	0.935	0.010
Sterol ester (27:1/22:6)	Weighted median	0.779	0.572	1.060	0.112
	MR-Egger	0.605	0.351	1.043	0.092
	Inverse variance weighted	0.691	0.495	0.965	0.030
Sterol ester (27:1/15:0)	Weighted median	0.751	0.481	1.171	0.206
	MR-Egger	0.851	0.475	1.524	0.607
	Inverse variance weighted	0.761	0.589	0.984	0.037
Phosphatidylethanolamine (O-18:2_20:4)	Weighted median	0.836	0.590	1.184	0.313
	MR-Egger	0.668	0.362	1.230	0.227
	Inverse variance weighted	0.864	0.753	0.992	0.039
Phosphatidylethanolamine (18:0_20:4)	Weighted median	0.896	0.750	1.070	0.224
	MR-Egger	0.904	0.644	1.269	0.570
	Inverse variance weighted	0.835	0.702	0.992	0.040
Sphingomyelin (d34:0)	Weighted median	0.842	0.665	1.066	0.152
	MR-Egger	0.891	0.661	1.199	0.457

### 3.2 Sensitivity analysis

According to the Cochran Q test, our IVW–MR analysis results demonstrated no evidence of heterogeneity among our reported results. The MR-Egger regression analysis results provided evidence that there was no other significant horizontal pleiotropy (**Table 2**). We also conducted the leave-one-out method to identify and delete abnormal instrumental variables. The results showed the robustness of our results (**Supplementary Figure S1**). These results suggest that the MR analysis results were relatively stable.

**Table 2:** Sensitivity analysis of plasma lipidome on rosacea.

Exposure	Q	*P*-value for Cochran Q test	Egger-intercept	*P*-value for MR-Egger intercept
Sterol ester (27:1/22:6)	14.612	0.405	0.040	0.395
Sterol ester (27:1/15:0)	3.326	0.767	−0.039	0.426
Phosphatidylethanolamine(O-18:2_20:4)	5.899	0.750	0.020	0.653
Phosphatidylethanolamine (18:0_20:4)	7.134	0.929	−0.012	0.780
Sphingomyelin (d34:0)	8.819	0.887	−0.013	0.607

## 4 Discussion

We conducted an MR analysis to investigate the causal relationship between plasma lipidome and rosacea utilizing GWAS summary-level data. Our results showed that five lipid species have negative causal relationship on rosacea, specifically, two species of sterol ester, two species of phosphatidylethanolamine, and one species of sphingomyelin. To the best of our knowledge, the current analysis of plasma lipidome on rosacea is limited, and the relationship between plasma lipidome and rosacea has not been reported.

Sterol ester, formed through the esterification of sterols and fatty acids, belongs to the sterols category, playing a crucial role in maintaining the structural and functional integrity of cellular membranes. They modulate membrane fluidity and stability, which in turn affect cellular responsiveness to external stimuli and signal transduction (20, 21). Generally, sterols are primarily taken up through dietary sources and synthesized in the liver. Additionally, cholesterol biosynthesis enzymes are expressed in primary and secondary lymphoid organs, which suggested that sterols play a role in immune regulation. Accordingly, systemic sterols modulate immune cell biology (22); furthermore, in inflammatory processes, sterol esters may act as signaling molecules or a regulatory agent (23). Meanwhile, our understanding of how sterols modulate specific immune cell biology is limited (20, 24).

Phosphatidylethanolamine (PE) is one of the most abundant phospholipids in plasma membranes (25). Besides being a passive membrane constituent, PE is also functionally associated with protein biogenesis and activity (26), oxidative phosphorylation (27), and autophagy (28) and is an important precursor of other lipids (29). The localization of PE changes during cell death. PE resides predominantly in the inner leaflet of the cell membrane in healthy cells; on the other hand, PE is externalized to the outer leaflet of the plasma membrane in dead or dying cells (25). It is demonstrated that PE is associated with Alzheimer’s and Parkinson’s disease and liver steatosis and steatohepatitis (29).

Sphingomyelin (SM) is one of the main phospholipids that make up the hydrophobic matrix of mammalian membranes, which are considered a “structural” lipid and contribute to the geometrical stability of the cell membranes (30). Recently, studies reported that the SM metabolic pathway contributes significantly to cell signaling, especially in regulating tumor cell growth, differentiation, senescence, and survival (31). Accordingly, SM acts as a critical molecule for brain physiopathology, playing a role in Parkinson’s disease progression (32). In addition, SM regulates cell growth, differentiation, and apoptosis in colorectal cancer and decreases colonic inflammation and inflammation-driven colorectal cancer (33).

Although no MR analysis reported the causal relationship between plasma lipidome and rosacea, some MR analyses reported the causal relationship between plasma lipidome and other illness. MR analysis revealed that phosphatidylinositol and triglyceride levels decreased the risk of breast cancer (BC) (34); genetically increased triglycerides were closely related to an elevated risk of Barrett’s esophagus (BE) (35).

Studies also reported the role of plasma lipidome in rosacea. Neutrophils and HDL, instead of LDL, have effects on the risk or severity of rosacea (36). Moreover, a meta-analysis performed on large groups of patients with rosacea and controls revealed that rosacea is significantly associated with dyslipidemia and higher total cholesterol, LDL, and triglyceride concentrations (37). The explanation for the association between rosacea and dyslipidemia is uncertain. Studies showed the activation of nucleotide binding oligomerization domain-like receptor 3, which can cause IL-1β release and induce structural changes of lipoproteins, decreasing their ability to break down and transport cholesterol (37, 38). An earlier study on the skin surface lipids in rosacea revealed that the lipid contents in the skin, particularly cholesterol, free fatty acids, triglycerides, esters, and squalene, were no different between patients and controls without rosacea (39). It should be noted that this research was performed on a small group of patients (N=31) and focused on skin lipidomics, not plasma lipidome. In actuality, research examining the roles of sterol esters, PE, and SM in the context of rosacea is relatively scarce.

Our research employed MR analysis to investigate the causal relationship between various plasma components and rosacea. The results suggest that sterol esters, PE, and SM may serve as protective factors against rosacea. Identifying novel biomarkers could enhance our understanding of the pathogenesis of rosacea and facilitate improved assessment of patients suffering from this dermatological condition. The implications of our findings may extend to both experimental design and clinical practice. Future investigations will focus on the roles of sterol esters, PE, and SM in rosacea, utilizing cell culture and animal models for further exploration.

There are several limitations to our study. First, due to the original GWAS statistics, we were unable to divide the cohorts or perform subgroup analyses. Second, our analysis only included individuals of the European population. Although using a single European population to investigate causal relationships can minimize population stratification bias, it is important to interpret these findings with caution regarding their applicability to other populations; in addition, there was no validation performed using a different set of data.

Our findings reported that sterol ester, PE, and SM have nominal causal connections with rosacea, but these correlations vanished after applying the FDR correction. It is important to note that the FDR correction can result in false negatives (40).

We have established a causal relationship between sterol ester, PE, and SM in relation to rosacea; however, the expression levels of specific liposomes in patients with rosacea remain uncertain. Furthermore, the underlying mechanisms by which these lipids exert their effects are not yet fully understood, necessitating further investigation. It is important to emphasize that while our study did not identify any associations between other subtypes of the plasma lipidome and the risk of rosacea, this absence of evidence does not imply that these other subtypes lack an influence on the condition. Our research serves as a hypothesis-generating endeavor for exploratory purposes.

## 5 Conclusion

In summary, our MR study presents evidence suggesting that sterol esters, phosphatidylethanolamine (PE), and sphingomyelin (SM) exert a negative causal influence on rosacea. This finding indicates that sterol esters, PE, and SM may play a protective role in the pathophysiology of rosacea. Future investigations into the plasma lipidome may yield innovative therapeutic targets and clinical strategies for the management of rosacea.

## Data Availability

The original contributions presented in the study are included in the article/**Supplementary Material**. Further inquiries can be directed to the corresponding author.

## Supplementary material

Supplementary Figure 1 Results of “leave-one-out” sensitivity analysis in the discovery and validation datasets. **(A)** Sterol ester (27:1/22:6). **(B)** Sterol ester (27:1/15:0). **(C)** Phosphatidylethanolamine (O-18:2_20:4). **(D)** Phosphatidylethanolamine (18:0_20:4). **(E)** Sphingomyelin (d34:0).
