# Supplementary figures and images for: Causal relationship between plasma lipidome and rosacea: a Mendelian randomization analysis
Source: Front Endocrinol (Lausanne). 2025 Apr 28;16:1427656. doi: 10.3389/fendo.2025.1427656 (PMC12066300; doi:10.3389/fendo.2025.1427656)

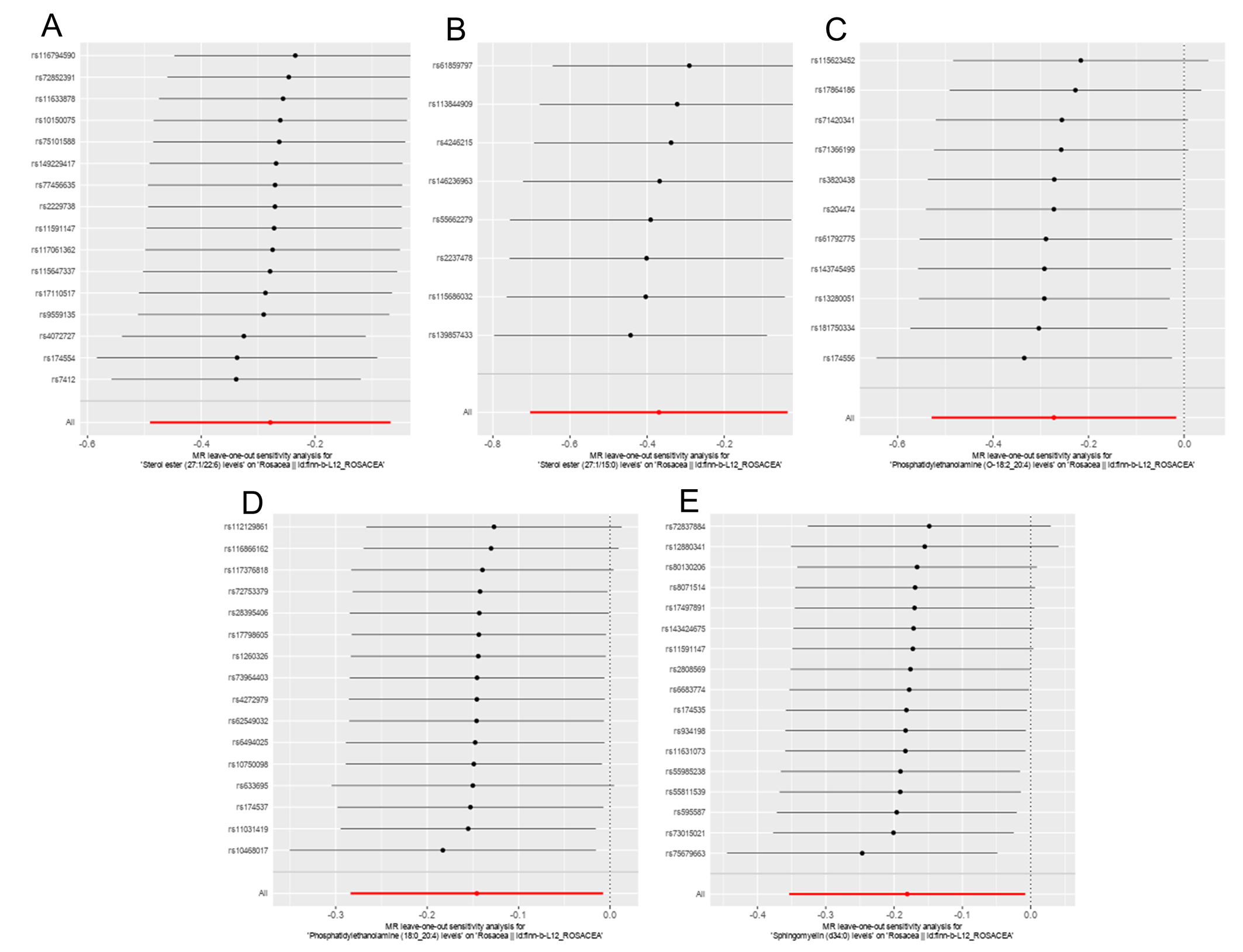

Supplement: Supplementary Figure 1 — Results of “leave-one-out” sensitivity analysis in the discovery and validation datasets. (A) Sterol ester (27:1/22:6). (B) Sterol ester (27:1/15:0). (C) Phosphatidylethanolamine (O-18:2_20:4). (D) Phosphatidylethanolamine (18:0_20:4). (E) Sphingomyelin (d34:0). [file Image1.tif]
